# Co-morbidity of personality disorder in schizophrenia among psychiatric outpatients in China: data from epidemiologic survey in a clinical population

**DOI:** 10.1186/s12888-016-0920-8

**Published:** 2016-07-08

**Authors:** YanYan Wei, TianHong Zhang, Annabelle Chow, YingYing Tang, LiHua Xu, YunFei Dai, XiaoHua Liu, Tong Su, Xiao Pan, Yi Cui, ZiQiang Li, KaiDa Jiang, ZePing Xiao, YunXiang Tang, JiJun Wang

**Affiliations:** Department of Medical Psychology, Faculty of Mental Health, Second Military Medical University, Shanghai, 200433 People’s Republic of China; Shanghai Mental Health Center, Shanghai Jiaotong University School of Medicine, 600 South Wanping Road, Shanghai, 200030 People’s Republic of China; Department of Psychological Medicine, Changi General Hospital, Singapore, Singapore; Shanghai Key Laboratory of Psychotic Disorders (No.13dz2260500), Bio-X Institutes, Key Laboratory for the Genetics of Developmental and Neuropsychiatric Disorders (Ministry of Education), Shanghai, People’s Republic of China

**Keywords:** Schizotypal personality disorder, Prevalence, Cluster-A personality disorders, Psychosis, Clinical population

## Abstract

**Background:**

The reported rates of personality disorder (PD) in subjects with schizophrenia (SZ) are quite varied across different countries, and less is known about the heterogeneity of PD among subjects with SZ. We examined the co-morbidity of PD among patients who are in the stable phase of SZ.

**Method:**

850 subjects were randomly sampled from patients diagnosed with SZ in psychiatric and psycho-counseling clinics at Shanghai Mental Health Center. Co-morbidity of PDs was assessed through preliminary screening and patients were administered several modules of the SCID-II. Evidence of heterogeneity was evaluated by comparing patients diagnosed with SZ with those who presented with either affective disorder or neurosis (ADN).

**Results:**

204 outpatients (24.0 %) in the stable phase of SZ met criteria for at least one type of DSM-IV PD. There was a higher prevalence of Cluster-A (odd and eccentric PD) and C (anxious and panic PD) PDs in SZ (around 12.0 %). The most prevalent PD was the paranoid subtype (7.65 %). Subjects with SZ were significantly more likely to have schizotypal PD (4.4 % vs. 2.1 %, *p* = 0.003) and paranoid PD (7.6 % vs. 5.4 %, *p* = 0.034), but much less likely to have borderline, obsessive-compulsive, depressive, narcissistic and histrionic PD.

**Conclusions:**

These findings suggest that DSM-IV PD is common in patients with SZ than in the general population. Patterns of co-morbidity with PDs in SZ are different from ADN.

## Background

There is a close relationship between personality disorder (PD) and schizophrenia (SZ). This relationship is supported by epidemiological, phenomenologic, and biologic studies [1–6]. However, patients with SZ do not appear to adhere to the normative pattern of co-morbidity. Research has supported the presence of co-morbidity of PD traits in subjects with SZ [7–9]. The presence of SZ traits may potentially have enormous impacts on a PD assessment. Despite the prevalence of co-morbidities in SZ, there is a relative dearth of information in the current literature of Chinese population. Moreover, estimates of the prevalence of PD are quite varied across epidemiological surveys in different countries. This ranges between 4.5 % and 100 % [10–12] among patients with SZ and other psychotic disorders.

Furthermore, former investigations present with limitations that may affect a comprehensive understanding of the rates of PD in patients with SZ. The epidemiological survey in this population in the past 2 decades showed no persuasive evidence of the co-morbidity between SZ and PD. This is in part due to inadequate sample sizes and non-representation of target sample. The demographic and clinical profiles could be a critical component in deciding whether there is difference on PD prevalence rates. Several previous studies contained mixed samples (e.g. including schizoaffective disorder and other forms of psychoses). Sample sizes were also generally small, and lacked the inclusion of various clusters and subtypes of PD. Hence it calls for further investigation to address the question of prevalence of PD in SZ.

Both PD and SZ are chronic and debilitating mental disorders. Hence, it is important to collect epidemiological data on the co-morbidity of PD and SZ [13–16]. This data will have practical implications for practicing clinicians [15, 17]. A recent study indicated that co-morbid Borderline PD has a significant negative longitudinal impact on the course and outcome of patients with SZ [18]. Examining PD in SZ patients will also help us understand the "schizophrenic spectrum" concept, which includes group disorders like SZ and Cluster A PDs (i.e. schizotypal, schizoid, and paranoid PD) that could be genetically inherited [19–21]. As mentioned in previous studies, some PDs traits may be an independent risk syndrome for psychosis [22, 23]. Hence, it is important to identify a possible range of pre-morbid PDs that may occur as part of the prodromal phase of SZ that addresses both symptoms and risks for future psychosis.

It has been recognised that pathological personality occurs in the context of SZ both before and after the onset of psychosis. Hence, several papers put forward the notion that some pre-morbid subtypes of PD (such as schizotypal PD) may be more vulnerable to SZ, and as a result, affect the prognosis of SZ. There is an abundance of evidence that supports the high co-morbidity rate between PD and affective or anxiety disorders [24–27]. Yet, compared to other mental disorders, literature on the co-morbidity of PD with SZ is significantly lesser. It appears that clinicians in China also tend to pay less attention to a co-morbid PD diagnosis with patients with SZ.

There are no existing reports on the prevalence of PD in Chinese patients with SZ. Even in the Chinese literature, related data of pathological personality in SZ are rare. Because diagnosis of PD in China adheres to the CCMD-3 standards (Chinese Classification and Diagnostic Criteria of Mental Disorders, Third Edition), other mental disorders recognised by the DSM-IV (Diagnostic and Statistical Manual of Mental Disorders, 4th Edition) are excluded. As a result, the co-morbidity of PDs in SZ in China remains largely unknown. The results of PD rates vary in different countries. For example, the Canadians and Swedish were less likely to meet a diagnosis of PD in the psychotic population compared to the Spanish patients [11, 28]. The diagnosis of PD may be influenced by cultural and social backgrounds. The Chinese grew up under the strong influence of traditional eastern values while facing rapid changes in the social structure. This has, no doubt, a broad range of impacts on the Chinese community, and hence is worth exploring this phenomenon.

The primary objective of the present study is to examine the distribution of PDs in Chinese patients with SZ. To the best of our knowledge, this is the first study that examines the prevalence of DSM-IV PD using a large clinical sample with SZ in China. It is also an examination of the extent of PD-SZ co-morbidity using DSM diagnoses compared to those with affective disorder or neurosis (ADN). A few hypotheses (such as the pre-morbid model for spectrum hypothesis and the post-morbid model for scar hypothesis) were tested in efforts to enhance our understanding of their inter-relations and differences. In addition, the current study is the first to discuss the feasibility and necessity in carrying out possible comprehensive interventions on pathological personality in SZ population.

## Method

### Subjects

The epidemiologic survey on PD in clinical settings was conducted in 2006 in Shanghai [29–33]. As detailed elsewhere [30], the participants were recruited from the largest psycho-counseling and psychiatric clinics in Shanghai. Two staff members issued invitation letter to the one-tenth outpatient in psycho-counseling and one-twentieth outpatient in psychiatric clinics according to hospital registration list. Thus, in total of 3402 subjects were randomly sampled from the mental health service setting. During the preliminarily screening process, 327 ineligible outpatients were excluded according to the inclusion and exclusion criteria. After obtaining the patients' informed consent, 3075 subjects were included in the study, and screened with a self-reported questionnaire (the Personality Diagnostic Questionnaire, PDQ-4+). Subsequently, 2590 participants who screened positive were referred for a face-to-face interview (Structured Clinical Interview for DSM-IV AxisII, SCID-II). The CCMD-3 diagnoses were collected according to outpatients’ medical records.

For this paper, we obtained data on a selected sample of outpatients with SZ. About 951 outpatients (30.9 %) were diagnosed with SZ or other psychotic disorders according to their medical records. To ensure that selection is only limited in SZ, clinicians were asked to check the patients’ medical records. 101 subjects (10.6 %) were then excluded because their diagnosis was unclear or other psychotic disorders (such as paranoid psychosis, acute transient psychosis, traveling psychosis et al.).

Consequently, the remaining 850 subjects with SZ who were recruited from the previous epidemiological survey between May and October 2006 were analyzed. Amongst them were 184 subjects (21.6 %) from the psycho-counseling clinic and 666 subjects (78.4 %) from the psychiatric clinic. There were 371 males (43.6 %) and 479 females (56.4 %). The average age was 31.7 years (SD = 9.8). The average course was 85.2 months (SD = 95.8) within the range of 1–480 month. 543 participants (63.9 %) were single, 238 (28.0 %) were married, 54 (6.4) were divorced, 15 (1.8 %) were widowhood; 323 (38.0 %) completed college or higher; while 453 (53.3 %) participants earned less than 1000 Yuan a month.

From these 3075 subjects, 1403 outpatients who were diagnosed with affective disorder or neurosis (ADN) were selected as the control group. They were identified through their medical records. Amongst those outpatients, there were 605 males and 798 females, with a mean age of 32.6 years (SD = 10.2, ranged 18–60 years). 742 subjects with mood disorders (including bipolar disorder and depression), 661 subjects with neurosis (including anxiety disorders (*N* = 517) such as phobia, panic disorder, generalized anxiety disorder, obsessive-compulsive disorder, and somatoform disorders (*N* = 144) such as somatization disorder, hypochondriasis, neurasthenia).

### Assessment tools of personality disorders and procedures

The Research Ethics Committee at the Shanghai Mental Health Centre approved the study in 2006. All participants were given detailed explanation about the study, including a plain language statement written in their native language. Their written informed consent was obtained before recruited for the study. Only participants of the clinic who were judged to be fully competent to give informed consent for participation were included. An independent senior psychiatric nurse judged if the individual with SZ was competent in giving informed consent. Participation could be withdrawn at any time, and non-participation in the research would not affect the quality of clinical care.

PD assessments were administered using the Personality Diagnostic Questionnaire (PDQ-4+) and Structured Clinical Interview for DSM-IV AxisII(SCID-II)[30]. Both correspond to the criteria of DSM-IV PD. The SCID-II is a semi-structured clinical interview for PD diagnosis. It contains 12 subscales corresponding to the 12 Axis II DSM-IV PDs, which comprise Cluster A PD (Paranoid, Schizoid, Schizotypal PD), Cluster B PD (Histrionic, Narcissistic, Borderline, Antisocial PD), Cluster C PD (Avoidant, Dependent, Obsessive-compulsive PD), Passive-aggressive PD and Depressive PD (in the appendix of DSM-IV). The sample was selected using a two-stage probability sample design. At the first stage, all the eligible subjects was screened by PDQ-4+, which conducted by three senior nurses. At the second stage, patients whose PDQ-4+ score met some form of PD subsequently attended the SCID-II interview by two trained psychiatrists. The Chinese version of SCID-II was previously translated and adapted by the research team members [34].

PDQ-4+ screening questions were administered to 850 subjects with SZ. PDQ-4+ has been widely used to screen for DSM-IV PD. Although specificity is medium, it is a relatively sensitive (0.89) test. 523 subjects whose PDQ-4+ test result was positive were interviewed with the SCID-II. The diagnosis of PDs requires evaluation of long-term patterns of functioning. Hence that implies that there is a potential for state effects. Subjects were asked to reflect and discuss about “everyday life” rather than “recent situations” during PD assessments.

### Diagnosis of Schizophrenia and other Axis I disorder

CCMD-3 (the Chinese Classification of Mental Disorders) is substantially influenced by ICD-10 and DSM-IV representations. The uniaxial classification system used in CCMD gave little thought to issues of co-occurring PDs with other disorders. Most of the diagnostic criteria of SZ are identical to international classifications. However, there is a major difference between the CCMD-3 and DSM systems in the diagnosis of SZ; the duration criterion for SZ is 1 month in CCMD-3, but 6 months in DSM-IV.

Subjects with ADN were considered as a control group because overwhelming clinical evidence showed that depression and neurosis frequently coexist in the same individual, either concurrently or at different times. Therefore, it is hard to distinguish mood and neurosis in detail. This group is also highly heterogeneous with SZ group. Differences include clinical characteristics, presence of predisposing personality, social factors, and the preservation of insight. Amongst the patients in psychiatric clinics and psycho-counseling clinics, SZ, affective disorders and neurosis made up the majority (making up nearly 3/4 of the total sample in 2006).

### Statistical analysis

All analyses presented were conducted for the sample with SZ and have used ADN as the comparative group. Frequencies and 95 % confidence interval (95 % CI) of PDs, in accordance to PDQ-4+ and SCID-II were calculated by cluster and specific PD. These comparisons assessed for unadjusted differences by age, sex, education and marriage state, proportions of patients visiting psycho-counseling or psychiatric clinic, self-reported characteristics and course. Odds ratios (OR) were generated to assess associations of PD with those socio-demographic characteristics. Before applying parametric statistics, all variables were checked the normality of the scores distribution.

Two tailed t-tests were used to compare PDQ-4+ mean scores of patients with SZ and ADN by cluster and specific PD. Chi-squared tests were used to compare the proportions of PD patients according to SCID-II. All statistical differences were considered significant at *P* < 0.05. Stepwise regression modeling was used to assess the impact of PD on a mental disorder diagnosis. The presence of SZ was entered as the dependant variable and age, sex and the presence of PD were entered as the independent variables. OR, 95 % CI, *χ*^*2*^ statistic and *p* value for all individual variables in the final model were estimated.

## Results

### Prevalence of PDs

When the self-rating tool of PDQ-4+ was used for described the feature of PDs, the rate of endorsement of PD traits in our schizophrenics sample with was relatively high (Table 1). 81.5 % participants presented with at least one PD traits. The most frequent PD trait in this sample was avoidant PD (52.6 %), followed by obsessive-compulsive (48.2 %) and paranoid PD (39.3 %).

**Table 1:** Frequency of DSM-IV PDs among patients with schizophrenia using PDQ-4+ and SCID-II

	PDQ-4+		SCID-II	
	*N* (%)	95 % CI	*N* (%)	95 % CI
^a^Any PDs	693 (81.53 %)	78.92 – 84.14 %	204 (24.00 %)	21.13 – 26.87 %
Any Cluster A PD	503 (59.18 %)	55.87 – 62.48 %	103 (12.12 %)	9.92 – 14.31 %
Paranoid PD (PAR)	334 (39.29 %)	36.01 – 42.58 %	65 (7.65 %)	5.86 – 9.43 %
Schizoid PD (SCH)	167 (19.65 %)	16.98 – 22.32 %	20 (2.35 %)	1.33 – 3.37 %
Schizotypal PD (SCHT)	314 (36.94 %)	33.70 – 40.19 %	37 (4.35 %)	2.98 – 5.72 %
Any Cluster B PD	424 (49.88 %)	46.52 – 53.24 %	32 (3.76 %)	2.49 – 5.04 %
Histrionic PD (HIS)	254 (29.88 %)	26.81 – 32.96 %	10 (1.18 %)	0.45 – 1.90 %
Narcissistic PD (NAR)	220 (25.88 %)	22.94 – 28.83 %	9 (1.06 %)	0.37 – 1.75 %
Borderline PD (BOR)	322 (37.88 %)	34.62 – 41.14 %	17 (2.00 %)	1.06 – 2.94 %
Antisocial PD (ANT)	215 (25.29 %)	22.37 – 28.22 %	2 (0.24 %)	0-0.56 %
Any Cluster C PD	580 (68.24 %)	65.11 – 71.37 %	102 (12.00 %)	9.82 – 14.18 %
Avoidant PD (AVO)	447 (52.59 %)	49.23 – 55.95 %	64 (7.53 %)	5.76 – 9.30 %
Dependent PD (DEP)	254 (29.88 %)	26.81 – 32.96 %	29 (3.41 %)	2.19 – 4.63 %
Obsessive-compulsive PD (OBC)	410 (48.24 %)	44.88 – 51.59 %	30 (3.53 %)	2.29 – 4.77 %
In the Appendix of DSM-IV				
Passive-aggressive PD (PAG)	292 (34.35 %)	31.16 – 37.55 %	23 (2.71 %)	1.62 – 3.80 %
Depressive PD (DPS)	259 (30.47 %)	27.38 – 33.56 %	44 (5.18 %)	3.69 – 6.67 %

^a^Only includes 12 PDs as stated in the DSM-IV, not include PD not otherwise specified (PD NOS)

When structured interview tool of SCID-II were used for PD diagnosis, the frequency of PD among respondents with SZ was also common. Nearly one quarter of participants meet criteria for at least one DSM-IV PD, with the most prevalent PD was paranoid PD (7.65 %), followed by avoidant (7.53 %) and schizotypal (4.35 %) PD. Cluster A PDs (12.12 %) were the most common PD types compared to other clusters.

### Association with clinical and socio-demographic characteristics

The overall prevalence of PDs was significantly greater in younger group than older (OR = 1.969) (Table 2). Subjects who were single (OR = 1.812), had a greater prevalence compared to married status. PDs were also significantly more prevalent in the psycho-counseling clinic (OR = 2.438) than in Psychiatric clinic. The prevalence of PD also varied across categories of self-reported characteristics (Introversion, Middle type, Extroversion). PDs were most prevalent in subjects who had characteristics of Introversion (OR = 1.982). Subjects who had an ill duration of lesser than 6 months were more prevalent compared to those with a more chronic presentation, but the odds of having PD did not differ significantly.

**Table 2:** Odds ratios (and 95 % CI) of having PDs, by clinical and socio-demographic characteristics

	Number	PD	PD (%)	95 % CI	OR	95 % CI
Male	371	86	23.2	18.9 ~ 27.5 %	0.923	0.671 - 1.269
Female	479	118	24.6	20.8 ~ 28.5 %	1.083	0.788 – 1.489
~30 Years	439	131	29.8	25.6 ~ 34.1 %	1.969	1.423 – 2.726
30 ~ Years	411	73	17.8	14.1 ~ 21.5 %	0.508	0.367 – 0.703
Middle or high school	527	127	24.1	20.4 ~ 27.8 %	1.014	0.733 – 1.403
College or higher	323	77	23.8	19.2 ~ 28.5 %	0.986	0.713 – 1.364
Single or divorced, widowhood	612	164	26.8	23.3 ~ 30.3 %	1.812	1.234 – 2.660
Married	238	40	16.8	12.1 ~ 21.6 %	0.552	0.376 – 0.810
Psycho-counseling clinic	184	70	38.0	31.0 ~ 45.1 %	2.438	1.713 – 3.469
Psychiatric clinic	666	134	20.1	17.1 ~ 23.2 %	0.410	0.288 – 0.584
Introversion	438	131	29.9	25.6 ~ 34.2 %	1.982	1.431 – 2.743
Middle type	293	53	18.1	13.7 ~ 22.5 %	0.594	0.418 – 0.844
Extroversion	119	20	16.8	10.1 ~ 23.5 %	0.601	0.361 – 0.999
Course < =6 month	151	45	29.8	22.5 ~ 37.1 %	1.442	0.975 – 2.132
Course >6 month	699	159	22.7	19.6 ~ 25.9 %	0.694	0.469 – 1.025

Note: Age grouped by median age of the sample

### Comparative analyses of personality disturbance

As seen in Table 3, the first set of analyses focused on the self-reported personality disturbance. Subjects with SZ reported significantly less symptoms of cluster B and C PD traits compared to subjects with ADN, except the dependent PD. The mean score of cluster A PDs of two groups did not differ significantly. The second set of analyses was conducted to investigate the difference of PD prevalence between the SZ and ADN group. Subjects with SZ were significantly more likely to have schizotypal PD (4.4 % vs. 2.1 %, *p* = 0.003) and paranoid PD (7.6 % vs. 5.4 %, *p* = 0.034), but much less likely to have borderline, obsessive-compulsive, depressive, narcissistic and histrionic PD.

**Table 3:** Comparison of difference between SZ and ADN for PDQ-4+ PDs scores and SCID-II PDs frequency

	PDQ-4+			SCID-II		
	SCH	ADN	*t* value	SCH (%)	ADN (%)	*χ* ^*2*^ value
Any Cluster A PD	9.04	9.04	−0.028^a^	12.1%	9.6 %	0.054
PAR	2.82	2.97	−1.759	7.6 %	5.4 %	0.034*
SCH	2.39	2.41	-.308^a^	2.4 %	2.6 %	0.677
SCHT	3.82	3.66	1.648	4.4 %	2.1 %	0.003**
Any Cluster B PD	12.32	13.05	−2.498^a^*	3.8 %	12.2 %	0.000**
HIS	3.34	3.64	−3.705**	1.2 %	3.0 %	0.005**
NAR	3.10	3.38	−3.084**	1.1%	2.9 %	0.004**
BOR	4.06	4.44	−3.472**	2.0 %	7.6 %	0.000**
ANT	1.82	1.60	3.155**	0.2 %	0.4 %	0.457
Any Cluster C PD	10.60	11.26	−3.105**	12.0 %	21.7 %	0.000**
AVO	3.61	3.89	−3.298**	7.5 %	9.8 %	0.063
DEP	3.32	3.41	-.998 ^a^	3.4 %	3.1 %	0.720
OBC	3.66	3.95	−3.467^a^ **	3.5 %	10.8 %	0.000**
In the Appendix of DSM-IV						
PAG	2.77	2.97	−2.740**	2.7 %	4.2 %	0.065
DPS	3.35	3.89	−5.987**	5.2 %	11.4 %	0.000**

^a^Levene's Test for Equality of Variances is significant; *p < 0.05; **p < 0.01

### Stepwise regression analyses

Stepwise regression was employed in an attempt to identify the risk factors of PDs related to SZ or ADN. Logistic regression (forward stepwise) analyses were performed in the presence of SZ as the dependent variable while age, gender, different type of PDs acted as independent variables (Table 4). Among those PDs, paranoid and schizotypal PD were significant predictors of SZ, and borderline, obsessive-compulsive, depressive, histrionic PDs were significant predictors of ADN.

**Table 4:** Forward stepwise logistic regression for risk factors predicting the clinical diagnoses

Variable	Beta	S.E.	OR	95 % CI	*χ* ^*2*^ statistic	*P* value
Age	0.011	0.005	1.011	1.003-1.020	6.335	0.012
PAR	−0.958	0.205	0.384	0.257-0.573	21.841	0.000
SCHT	−0.705	0.271	0.494	0.291-0.839	6.800	0.009
BOR	1.457	0.284	4.293	2.461-7.491	26.320	0.000
HIS	0.864	0.362	2.373	1.168-4.821	5.704	0.017
OBC	1.307	0.214	3.695	2.427-5.625	37.134	0.000
DPS	0.761	0.186	2.140	1.486-3.082	16.715	0.000
Constant	−0.006	0.155	0.994	-	0.002	0.967

## Discussion

SZ has always been considered the most serious mental illness in China, and usually accompanied with stigma or fear from the general community. The personality of this population is indeed difficult to understand and hence, is often neglected by people around them. Even psychiatric professionals only identify with symptoms such as hallucinations and delusions, and little attention has been given to the co-occurrence of PDs in the clinical assessment routine. This study is a groundbreaking survey of PDs among patients with SZ in the Chinese population. More than 80 % of subjects presented with at least one PD trait, and about one quarter of outpatients suffer from at least one diagnosable PD. Substantial evidence suggests that PDs influence other mental disorders’ prognosis, hence to a certain degree, overlook the PD in patients with SZ might missed the chance of predicting an Axis I prognosis and treatment response. Consequently, opportunities of a focused approach to treatment using psychotherapy are lost.

The prevalence of PD was 24.0 % in this Chinese clinical population with SZ, and was within the range of estimates (22 - 28 %) found in some previous epidemiologic surveys [35–37], but not in others [7, 38–40]. Newton-Howes and colleagues (2008) used the multilevel modeling method and found that there was great variation in the reported prevalence of PD in SZ. The multilevel modeling method identified that the most possible reason for this difference was that these studies were carried out in different countries. Other possibilities that may also have contributed to the discrepancies included sample size, differences in diagnostic criteria, assessment instruments, survey designs and methodologies.

After the SCID-II interview, Cluster A PDs (especially Paranoid PD) became the most prevalent PD compared to other PDs. That is, this pattern of PD co-morbidity may be explained by the spectrum hypothesis. Kraepelin and Bleuler considered schizophrenia as the worsening of schizoid features in the early 20th century. In the late 20th-century, Cluster A PDs was considered as SZ spectrum PDs [41–43]. It is also well known that Cluster A PDs was more likely to be noticed among relatives of patients with SZ [44, 45]. In addition, Cluster C PDs were also very common among those subjects. The finding that self-reported Cluster C PDs pathology (especially Avoidant and Obsessive-compulsive PD) is more common in Chinese patients with SZ is also consistent with the results of previous studies [28, 46]. This may be partly due to the effects of SZ on personality over time (the scar hypothesis). The experiences of stigma and social disability might account for ‘fearful’ group traits. This paper also analyzed the socio-demographic characteristics of patients with SZ. Taken as a whole, the factors associated with increased odds of PD were younger age, non-marriage status (single, divorced, or widowhood), in psycho-counseling clinic, and self-reported characteristics of introversion. This was consistent with findings from many prior studies, which identified that PDs were more prevalent among younger adults than older adults [47–50]. Although PDs are defined as stable and enduring patterns in the DSM, many previous PD surveys proposed that PD traits (especially Borderline PD traits) remit with age [51, 52]. Thus, from another viewpoint, this phenomenon is partly supported the scar hypothesis, which emphasized the influence of the presence of SZ on the features in personality psychopathology across the lifespan. Other characteristics might reflect that those schizophrenic patients with PDs live a solitary life, required more support and attention from the society, family and mental health professionals.

When compared to the patients with ADN, we found a distinct high co-morbidity of ‘eccentric’ group PDs amongst patients with SZ, while ‘emotional’ and ‘fearful’ group PDs were less commonly co-morbid. Paranoid and schizotypal PD seemed to be strongly associated with SZ, but borderline, obsessive-compulsive, depressive, histrionic PD were predictive of ADN. The results of this study showed that PD plays an important role in accurately diagnosing Axis I disorder. Because this was not a longitudinal study, it was difficult to determine the relation of consequences to antecedent. Hence, we mainly considered several possibilities with regard to the different pathological features of PDs among Axis I disorders: 1) certain cluster of pre-morbid PD may be earlier expressions of symptomatology in Axis I disorders; 2) certain cluster of pre-morbid PD may be vulnerabilities to SZ or ADN; 3) certain cluster of co-morbid PD may be the effects of the presence of Axis I disorders on personality over time.

The pattern of co-morbidity and heterogeneity of PD in SZ, as reported in this paper, may have several impacts on clinical practice. For example, it highlights the need for Chinese psychiatrists to pay attention to the following points. Firstly, as approximately one quarter of the recruited sample had been diagnosed with PD, detailed PD assessments and interventions should be considered for patients with SZ. That information will also be used to help us to improve the effectiveness and efficacy of the care provided at mental health service. Secondly, high comorbid rates of Cluster A PDs in SZ imply that subjects who fall in the schizophrenic spectrum PD may be strongly linked to SZ. Therefore, clinicians need to give attention to Cluster A PD-oriented therapy in order to minimize the risk of psychosis and long term sequelae. Lastly, the difference of comorbid PD between SZ and ADN suggests that there may be different ways of developing therapeutic alliance during therapy. Help-seeking behavior and medical resource utilization may be quite different with personality types. Therefore, a range of mental health services and social support may help meet the different needs for prevention and rehabilitation.

Several methodological limitations must be considered in regards to this study. The principal limitation of the cross-sectional design lies in its inability to distinguish between cause and effect of PD and SZ. The diagnosis of SZ in the study was made according to CCMD-3, which may then result in a fundamental difference in results of this sample with other international studies. PD symptoms, somehow, are a part of the syndrome of SZ, which may affect the accuracy of personality assessment. Subjects in this study were outpatients of the largest mental health service in Shanghai and therefore, this may introduce selection bias and reduce the generalizability of our findings to the general SZ population (i.e., recruitment from a general psychiatric hospital or forensic psychiatric hospital).

## Conclusions

Despite these limitations, these data provide an important step in understanding the rates of PDs among Chinese patients with SZ, as well as the association between PD and SZ. Furthermore, it helps in understanding the role of PD plays in the difference between SZ and ADN. Future research can consider following up with patients diagnosed with PD to ascertain how they are capable of acting on or influencing each other.

## Abbreviations

ADN, affective disorder or neurosis; ANT, antisocial PD; AVO, avoidant PD; BOR, borderline PD; CCMD-3, chinese classification and diagnostic criteria of mental disorders, Third Edition; DEP, dependent PD; DPS, depressive PD; DSM-IV, diagnostic and statistical manual of mental disorders, 4th Edition; HIS, histrionic PD; NAR, narcissistic PD; OBC, obsessive-compulsive PD; OR, odds ratio; PAG, passive-aggressive PD; PAR, paranoid PD; PD, personality disorder; PDQ-4+, personality diagnostic questionnaire, 4+ Edition; SCH, schizoid PD; SCHT, schizotypal PD; SCID-II, structured clinical interview for DSM-IV AxisII; SZ, schizophrenia.
